# Образовательные траектории эндокринологической службы: результаты анкетирования

**DOI:** 10.14341/probl13317

**Published:** 2023-06-30

**Authors:** Е. А. Пигарова, С. Ю. Воротникова, Т. Б. Белоцерковская

**Affiliations:** Национальный медицинский исследовательский центр эндокринологии; Национальный медицинский исследовательский центр эндокринологии; Национальный медицинский исследовательский центр эндокринологии

**Keywords:** медицинское образование, подготовка кадров, эндокринологическая служба, опрос, анкетирование, дополнительное профессиональное образование

## Abstract

Учитывая социально-экономические, политические и эпидемиологические изменения в субъектах Российской Федерации, вопрос модернизации подготовки медицинских кадров и планирования обеспечения медицинских организаций квалифицированными специалистами остается крайне важным в течение нескольких лет. Проведенный анализ анкетирования показал, что в настоящее время высокоактуальный вопрос кадрового обеспечения населения врачами-эндокринологами и врачами детскими эндокринологами не имеет однозначного эффективного решения. Приведенные меры по реорганизации образовательной среды требуют дополнительного пересмотра и коррекции для наиболее результативной работы сферы медицинского образования. В конечном итоге данные мероприятия скажутся на повышении уровня подготовки специалистов, улучшении кадровой ситуации в субъектах Российской Федерации и повышении качества оказания медицинской помощи населению страны.

## Abstract (en)

Given the socio-economic, political and epidemiological changes in the regions of the Russian Federation, the issue of modernizing the training of medical personnel and planning to provide medical organizations with qualified specialists has remained extremely important for several years. The analysis of the survey showed that at present the highly topical issue of staffing the population with endocrinologists and pediatric endocrinologists does not have an unambiguous effective solution. The above measures to reorganize the educational environment require additional revision and correction for the most effective work in the field of medical education. Ultimately, these activities will have an impact on improving the level of training of specialists, improving the personnel situation in the constituent entities of the Russian Federation and improving the quality of medical care for the population of the country.

С учетом социально-экономических, политических и эпидемиологических изменений в субъектах Российской Федерации вопрос модернизации подготовки медицинских кадров и планирования обеспечения медицинских организаций квалифицированными специалистами остается крайне важным в течение нескольких лет. Современные тенденции диктуют необходимость пересмотра образовательных стандартов в сфере здравоохранения как при подготовке врачей первичного звена, так и среднего медицинского персонала ввиду сохраняющегося кадрового дефицита [1]. Эндокринологическая служба Российской Федерации в настоящее время претерпевает ряд изменений, направленных на улучшение обеспечения населения врачами-эндокринологами и врачами детскими эндокринологами, а также повышение качества оказываемой помощи взрослому и детскому населению.

Недостаточная укомплектованность регионов врачами-эндокринологами и врачами детскими эндокринологами имеет различные причины [2]. Несомненно, качество высшего и среднего медицинского образования, система профессиональной переподготовки и повышения квалификации в рамках непрерывного медицинского образования являются одними из основополагающих факторов, потенциально позволяющих решить проблему кадрового обеспечения работниками здравоохранения в целом и профильными врачами в частности [3].

Анализ кадровой ситуации по профилю «эндокринология/детская эндокринология» проводится на основе оценки штатного состава региона в разрезе выделенных и занятых ставок врачей-эндокринологов и врачей-детских эндокринологов, а также количества физических лиц с использованием нормативных данных федеральной службы государственной статистики (РОССТАТ) статистических форм №30. По данным РОССТАТ, дефицит ставок врачей-эндокринологов наблюдался на начало 2022 г. в 55 регионах, суммарно 930,83 ставки, при этом дефицит ставок врачей детских эндокринологов — в 74 регионах, суммарно 411,5 ставки.

В целях определения возможных путей модернизации образовательной системы и улучшения качества обучения врачей первичного звена, врачей-специалистов и среднего медицинского персонала проведены сбор и анализ мнения работников здравоохранения в субъектах РФ о нынешней ситуации в образовательной среде. В рамках данного исследования разработан и предложен участникам анкетный опрос, позволяющий определить основные проблемы профессионально-квалификационного потенциала медицинских работников и возможные пути решения выявленных проблем. В опросе приняли участие 67 человек. Чуть более половины из них (50,7%) — работники высших учебных заведений, 32,8% — врачи государственных клиник, 9% — врачи государственных поликлиник, основными местами работы остальных участников опроса стали НИИ, частные медицинские организации. По основной специальности подавляющее количество респондентов — врачи-эндокринологи (97%), врачи детские эндокринологи (3%). Распределение респондентов по возрастам представлено на рис. 1.

**Figure:**
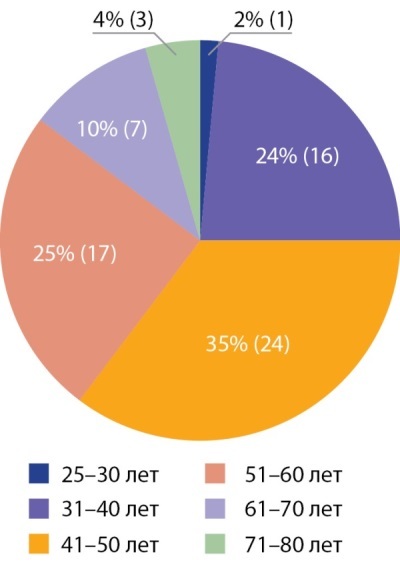
Рисунок 1. Возрастной состав участников опроса.Figure 1. Age composition of survey participants

Согласно полученным данным, большинство респондентов считают недостаточным уровень подготовки выпускников специалитета по вопросам эндокринологии как с теоретической, так и с практической (46%) стороны, примерно равное количество подчеркнули, что существует нехватка практических знаний в эндокринологии (38%), 13% человек посчитали, что подготовка кадров по программам специалитета в области эндокринологии достаточная, остальные участники опроса крайне не удовлетворены уровнем подготовки выпускников специалитета (рис. 2).

**Figure:**
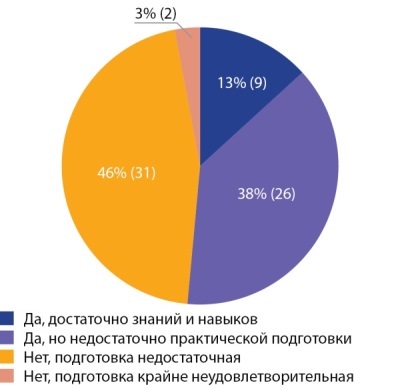
Рисунок 2. Достаточность объема знаний и практических навыков у выпускников медицинских образовательных учреждений.Figure 2. Sufficiency of the volume of knowledge and practical skills of graduates of medical educational institutions

Более 85% опрошенных считают необходимым изучение в рамках освоения программы специалитета таких тематик, как сахарный диабет 1 и 2 типа, патология щитовидной железы, патология гипофиза и гипоталамуса, патология надпочечников, нарушения фосфорно-кальциевого обмена, ожирение, а также неотложные состояния в эндокринологии (рис. 3).

**Figure:**
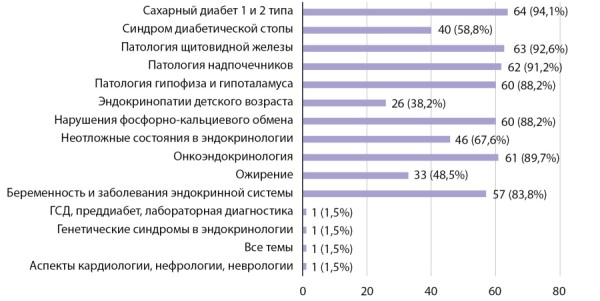
Рисунок 3. Тематики эндокринологии обязательные к изучению в рамках подготовки по программе специалитета.Figure 3. Subjects of endocrinology required to study in the framework of the preparation for the specialist's program.

Говоря о программах ординатуры по специальностям «Терапия» и «Общая врачебная практика», большинство респондентов (почти 90%) считают необходимым включение эндокринологии как отдельной дисциплины для изучения, при этом практически половина из них нашли оптимальным объем изучения «Эндокринологии» 144 и более академических часов. Практически аналогичным образом распределились мнения относительно наполнения дисциплины в рамках освоения программ ординатуры, чуть менее 20% участников посчитали необходимым включение тем о наследственных эндокринопатиях и редких формах сахарного диабета, остальные тематики, по мнению отвечавших, сходны с таковыми в рамках освоения программ специалитета.

Немаловажной составляющей высокого качества оказываемой пациентам с эндокринной патологией помощи ввиду широкой распространенности заболеваний является функционирование «Школ сахарного диабета». В этой связи опрос содержал ряд вопросов, касающихся медицинского персонала, осуществляющего работу такой структуры. Так, в ходе опроса выяснено, что, по мнению анкетируемых, обучение пациентов в «Школе сахарного диабета» должен осуществлять врач-эндокринолог, прошедший курсы повышения квалификации по соответствующей тематике длительностью не менее 36 академических часов (46% опрошенных). При этом практически половина (47%) считают необходимым для врача-эндокринолога обучение в рамках повышения квалификации по программе «Помповая инсулинотерапия» в объеме 36 академических часов, в то время как 25% нашли необходимым объем обучения по данной тематике в размере не менее 72 ч (рис. 4).

**Figure:**
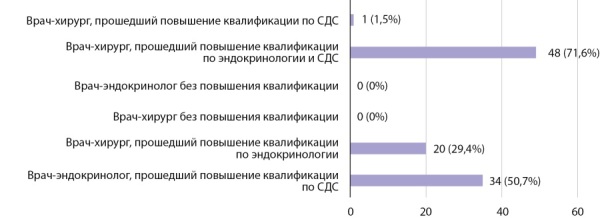
Рисунок 4. Оптимальный уровень подготовки врача кабинета «Школа для пациентов с сахарным диабетом».Figure 4. The optimal level of training of a doctor in the office "School for Patients with Diabetes"

Отмечено, что большинство участников анкетного опроса находят возможным проведение части занятий с пациентами в «Школе сахарного диабета» медицинской сестрой, прошедшей соответствующее обучение. По вопросу необходимого объема подготовки медицинской сестры мнения разделились следующим образом: 34% сочли необходимым изучать данный вопрос в течение 36 ч, практически такое же количество участников посчитали оптимальным объем 72 академических часа, почти 18% респондентов не видят необходимости в подготовке медицинской сестры «Школы сахарного диабета» более 18 ч.

Помощь пациентам, имеющим осложнения сахарного диабета, является неотъемлемой частью работы эндокринологической службы, а потому вопросы организации функционирования кабинетов «Диабетической стопы» очень актуальны. Так, участниками опроса было определено, что прием в кабинете «Диабетической стопы» должны вести либо врач-эндокринолог, либо врач-хирург, прошедшие курсы повышения квалификации по соответствующей теме, при этом обучение должно быть длительностью 36–72 академических часа, по мнению большинства участников анкетирования (рис. 5).

**Figure:**
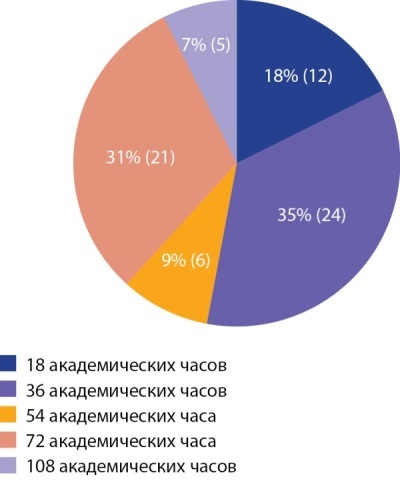
Рисунок 5. Необходимая длительность обучения в рамках повышения квалификации медицинских сестер по программе «Школа для пациентов с сахарным диабетом».Figure 5. Required duration of training as part of the advanced training of nurses under the program "School for Patients with Diabetes".

Несмотря на проводимые в сфере медицинского образования реформы, а именно увеличение доли целевых мест для обучения по программам ординатуры, в том числе по специальностям «Эндокринология» и «Детская эндокринология», данная мера не является универсальной для решения проблемы недостаточной укомплектованности как амбулаторного, так и стационарного звеньев врачами-эндокринологами и врачами детскими эндокринологами. Исходя из результатов опроса, 16,4% анкетируемых считают такой способ ликвидации кадрового дефицита вовсе неэффективным, в то время как 43,3% отметили частичную эффективность вышеуказанных мероприятий, а 40,3% опрошенных допускают возможность решения вопроса обеспеченности населения кадрами таким способом. При этом большинством голосов определен срок «отработки» от 3 до 5 лет наиболее оптимальным (рис. 6).

**Figure:**
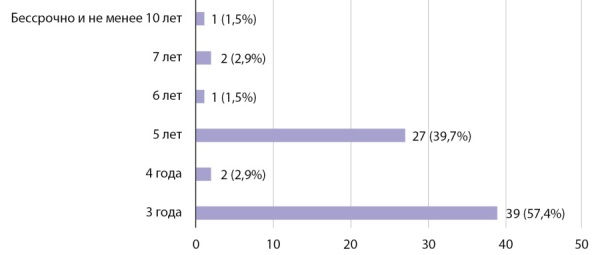
Рисунок 6. Длительность периода трудоустройства по целевому договору.Figure 6. Duration of the employment period under the target contract

Кроме того, респондентам анкетного опроса было предложено определить, какие структуры должны принимать участие в осуществлении отбора кандидатур на прохождение обучения в рамках целевого договора, а также структуры, осуществляющие контроль трудоустройства выпускников, обучавшихся по целевому договору. Более половины респондентов сочли необходимым участие Министерства здравоохранения или Департамента здравоохранения региона в данной процедуре, а также непосредственно медицинской организации, с которой был заключен договор. Практически половина (47%) посчитали важным участие главного внештатного специалиста региона в отборе кандидатов на обучение в ординатуре по специальностям «Эндокринология» и «Детская эндокринология» в рамках целевого договора (рис. 7).

**Figure:**
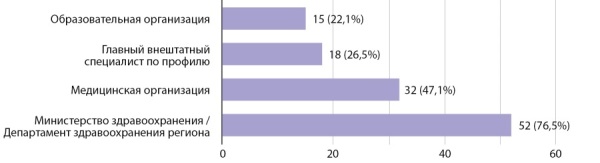
Рисунок 7. Структуры, которые должны контролировать трудоустройства выпускников по целевому договору.Figure 7. Structures that should control the employment of graduates under the target agreement

Таким образом, проведенный анализ показал, что в настоящее время крайне актуальный вопрос о кадровом обеспечении населения врачами-эндокринологами и врачами детскими эндокринологами не имеет однозначного эффективного решения. Кроме того, крайне важным было изучение мнения непосредственно работников здравоохранения, поскольку именно они являются ключевыми фигурами в данных вопросах. Очевидно, что проведенные меры по реорганизации образовательной среды требуют дополнительного пересмотра и коррекции для наиболее результативной работы сферы образования. В конечном итоге данные мероприятия направлены на повышение уровня подготовки специалистов, улучшение кадровой ситуации в субъектах Российской Федерации и повышение качества оказания медицинской помощи населению страны.

## ДОПОЛНИТЕЛЬНАЯ ИНФОРМАЦИЯ

Источники финансирования. Работа выполнена по инициативе авторов без привлечения финансирования.

Конфликт интересов. Авторы декларируют отсутствие явных и потенциальных конфликтов интересов, связанных с содержанием настоящей статьи

Участие авторов. Все авторы одобрили финальную версию статьи перед публикацией, выразили согласие нести ответственность за все аспекты работы, подразумевающую надлежащее изучение и решение вопросов, связанных с точностью или добросовестностью любой части работы.
